# From tokenism to transformation: lessons from the TOGETHER study for building inclusive and equitable research

**DOI:** 10.1186/s40900-026-00871-y

**Published:** 2026-03-30

**Authors:** Anita Mehay, Leandra Box, Karlet Manning, Annemarie Lodder, Trupti Balkrishna Patel, Donna Clutterbuck, Jabeer Butt, Richard G. Watt

**Affiliations:** 1 School of Health & Medical Sciences, City St George’s, University of London, Northampton Square, London, EC1V 0HB UK; 2 Race Equality Foundation, Deane House Studios, 27 Greenwood Place, London, NW5 1LB UK; 3 Public Involvement Contributor, London, UK; 4 School of Public Health, Imperial College London, White City Campus, 90 Wood Lane, London, W12 0BZ UK; 5 Sciencewise, Oxford House, Derbyshire Street, London, E2 6HG UK; 6 School of Primary Care, Population Sciences and Medical Education, University of Southampton, Southampton, SO17 1BJ UK; 7 Department of Epidemiology and Public Health, University College London, London, UK

**Keywords:** Health inequalities, Inclusion, Public involvement, Equity, PPIE, EDI, RCT, Community engagement, Parenting, Third sector

## Abstract

**Background:**

Children’s health in the UK is in decline, with widening inequities disproportionately affecting racially and socially minoritised families. These same communities are often excluded from research, compromising both fairness and scientific validity. Patient and public involvement and engagement (PPIE) has been promoted as a mechanism to address exclusion, but in practice it can replicate existing inequalities. There is limited evidence on what inclusive research looks like in practice. This paper reflects on the TOGETHER study—a large, multi-site randomised controlled trial of the *Strengthening Families*,* Strengthening Communities* (SFSC) parenting programme—exploring the strategies and processes that supported equitable engagement.

**Methods:**

We used a reflective, retrospective case approach informed by: (i) descriptive analysis of trial baseline data (recruitment, retention, participant demographics); (ii) analysis of five years of study meeting minutes; and (iii) two facilitated reflective workshops with parent advisory groups, the lived-experience co-investigator, and the Race Equality Foundation (third-sector partner).

**Results:**

The trial successfully recruited 674 parents across 34 programmes, meeting 100% of the target. The sample was both ethnically and socially diverse: 65% of participants identified as Black, Asian, mixed or other minoritised ethnicities; nearly half reported a first language other than English; and over half live with household incomes below £20,000. Attrition rates were 28% at post-intervention and 30% at six-month follow-up. Six key enablers were identified: (1) lived experience leadership through a co-investigator; (2) public involvement via local Parent Advisory Groups (3) relational partnerships with community organisations; (4) multilingual community researchers supporting linguistically and culturally inclusive data collection (5) strategic support from a third sector organisation, the Race Equality Foundation, and (6) investing in inclusion through dedicated budgets, resources, and visible, supportive leadership. These enablers helped ensure high recruitment, strong retention, and meaningful participation with families often excluded from research.

**Conclusions:**

The TOGETHER study demonstrates that inclusive research is possible when lived experience, community voices, and third-sector expertise are embedded and resourced from the outset. Inclusion required investment of time, money, and infrastructure, as well as leadership that valued relationships and reflexivity and researchers positioned not as detached observers but as relational actors within participants lived contexts. Our reflections highlight the potential and the tensions of embedding equity in research, offering practical insights for researchers, funders, and institutions seeking to move beyond tokenism towards transformation.

**Supplementary Information:**

The online version contains supplementary material available at 10.1186/s40900-026-00871-y.

## Abstract (plain language summary)

Children’s health in the UK is getting worse, with rising inequalities that particularly affect racially and socially minoritised families. Yet these families are often left out of research. This is unfair and also means that research findings may not apply to those who need them most. We looked back at the TOGETHER study, a large trial of the *Strengthening Families*,* Strengthening Communities* (SFSC) parenting programme, run in six areas of England. Our aim was to understand what made the study inclusive and how we involved parents and communities in meaningful ways. We analysed information on who joined the trial and who stayed involved and reviewed five years of meeting notes from the study team and public involvement groups. We also held reflection workshops and conversations with parents in the study advisory groups, a lived-experience co-investigator, and the Race Equality Foundation, our third-sector partner. The study recruited 674 parents across 34 programmes, meeting the initial study target. The parents were very diverse: 65% were from racially minoritised backgrounds, nearly half spoke a first language other than English, and over half lived in low household incomes. We identified six things that enabled an inclusive study: (1) leadership from lived experiencoe; (2) active parent advisory groups; (3)meaningful community partnerships; (4) community-embedded researchers; (5) the Race Equality Foundation linking researchers and communities; and (6) budgets, resources, and committed leadership. The study showed that inclusive research is not separate from people’s everyday lives. During the COVID-19 pandemic, for example, parents and researchers worked together to create accessible public health newsletters to support families. The TOGETHER study shows that inclusive research is possible when communities are embedded from the start and supported with the right resources.

**Supplementary Information:**

The online version contains supplementary material available at 10.1186/s40900-026-00871-y.

## Background

Improving children’s health is a vital foundation for lifelong wellbeing and population health [1]. Yet in the UK, children’s health is in worrying decline. One in five children now experiences a probable mental health disorder [2], and over 4.5 million live in poverty, with racially minoritised families disproportionately affected [3]. These stark inequities are not simply the result of individual choices but are shaped by the wider determinants of health, the conditions in which people are born, grow, live, work, and age [4]. For ethnically and socially diverse communities, these conditions are often defined by entrenched inequalities [5]. Yet these same communities are often excluded from the health, social care, policy and research systems meant to understand and address their needs—including research.

Health research plays a vital role in generating evidence to inform policy, drive innovation, and improve population outcomes [6]. However, ethnically and socially diverse populations remain persistently underrepresented in health research [7–10]. In the UK, an analysis of 148 research trials published by the National Institute for Health and Care Research (NIHR) between 2019 and 2021 found that 86% of participants were White [10]. Just 4% were Black and 5% Asian, figures that fall short of reflecting the ethnic diversity of the population. Only 60% of trials even reported participants’ ethnicity. Underrepresentation extends beyond recruitment and many studies also struggle to retain participants from minoritised groups, further threatening the validity and relevance of findings [11]. To understand what works to reduce inequalities, we cannot continue to study only the privileged.

The reasons for underrepresentation in research are complex and deeply embedded in structural and institutional systems [12]. Historical and contemporary experiences of exclusion, exploitation, and racism have led to deep mistrust of research institutions [13]. Language barriers, inflexible protocols, and inaccessible recruitment strategies continue to marginalise communities. People from ethnic minority backgrounds report previous negative experiences with health or social services, reinforcing a sense of alienation [8]. The consequences of this exclusion are about both fairness and scientific rigor. From an ethical perspective, the exclusion of ethnically and socially diverse communities from research limits whose experiences count and whose needs are prioritised. From a scientific perspective, research based on unrepresentative samples produces evidence that is narrow, non-generalisable, and potentially ineffective for the communities most affected. Inclusive research is therefore not simply about diversity for its own sake—it is essential to producing actionable, equitable evidence.

Patient and Public Involvement and Engagement (PPIE)—research carried out *with or by* members of the public rather than *to*, *about*, or *for* them—is now widely recognised as a core element of high-quality research [14–16]. Done well, it can challenge assumptions, reshape priorities, and improve research design and representation. It is also increasingly seen as a way to build trust and address exclusion—particularly for underserved or racially minoritised communities [17]. Yet PPIE is not immune to the inequalities it seeks to address. Public contributors remain largely drawn from narrow demographics—most often older, White, middle-class, and professional [18]. Diversity in PPIE panels is rarely reported, and groups facing the greatest health inequalities are often least likely to be included in PPIE [19]. As a result, PPIE that is not intentionally structured to include racially minoritised and socioeconomically disadvantaged communities risks reproducing existing inequities within research systems [7, 20].

Many of the barriers faced by racially minoritised and socioeconomically disadvantaged groups, such as discrimination, mistrust of institutions, and structural disadvantages [4, 5, 21], are reproduced within research systems [7, 20, 22, 23]. These same barriers affect both research participation and involvement in PPIE roles and activities. One review found that lack of trust, poor communication, and the dismissal of cultural concerns were key obstacles for racially minoritised communities [7]. Others experience digital exclusion or receive inadequate support for cognitive or communication needs, while many struggle to balance PPIE roles with complex personal, work, and caregiving responsibilities [20]. Some contributors also carry the invisible emotional labour of representing communities under strain—often without adequate recognition or support [24]. While some of these challenges stem from time and resource constraints, they also reveal a deeper lack of understanding among researchers about the lived realities and needs of diverse contributors [20].

These gaps point to a wider structural problem. PPIE is frequently promoted as a solution to exclusion, yet a growing body of literature highlights concerns about tokenism, performative engagement, and the reproduction of existing power hierarchies within research systems [22, 25]. Without intentional strategies to address structural inequities, PPIE risks becoming a procedural requirement rather than a transformative practice. Similar critiques have been raised in relation to diversity in clinical trials, where recruitment targets may be pursued without addressing the underlying relational and structural barriers to equitable participation [13, 25, 26]. To move beyond symbolic inclusion, we need clearer evidence of what inclusive research looks like in practice [8, 14]. Real-world case studies offer valuable insights into the challenges, tensions, and enablers of inclusion. Rather than relying on generalised commitments to diversity, researchers and funders must examine the mechanisms that underpin effective community engagement. This paper explores how inclusive research was realised in practice through PPIE and other strategies in the TOGETHER study—a large-scale trial of the Strengthening Families, Strengthening Communities (SFSC) parenting programme (funded by the NIHR).

## Overview of the TOGETHER study

The TOGETHER study was a national, multi-centre randomised controlled trial (RCT) [27] —widely regarded as the most rigorous design for evaluating the effectiveness of interventions and is frequently prioritised within evidence hierarchies [28]. RCTs randomly allocate participants to an intervention or control group, allowing for rigorous causal inference while minimising bias. RCT evidence is frequently prioritised within funding and policy/commissioning decisions so plays an influential role in determining which interventions are adopted, scaled or discontinued. This makes RCTs particularly important in the context of inequality where if trials do not adequately include racially and socioeconomically marginalised groups, the evidence base guiding policy may systematically overlook those most affected. The issue is therefore how we can design and deliver RCTs in ways that ensure inclusive participation and equitable representation.

The TOGETHER study set out to evaluate the effectiveness and cost-effectiveness of the *Strengthening Families*,* Strengthening Communities* (SFSC) parenting programme in improving parental mental well-being and children’s social and emotional development up to six months post-intervention [27]. The SFSC programme is a universal, evidence-informed programme deployed by the Race Equality Foundation—a third-sector organisation dedicated to race equity. Designed to meet the needs of racially and culturally diverse families, particularly Black, Asian, and minoritised ethnic communities, SFSC focuses on promoting protective parenting factors, positive behaviour change, and stronger community ties. While systematic reviews have shown that universal parenting programmes can support parenting skills, parental wellbeing, and child behaviour [29–31], such programmes can be less effective at engaging ethnically and socially diverse families [32]. SFSC stands out for its inclusive, community-focused model, and earlier evaluations have shown promising outcomes—but large-scale trials assessing its impact across diverse groups have been lacking.

**Fig. 1:**
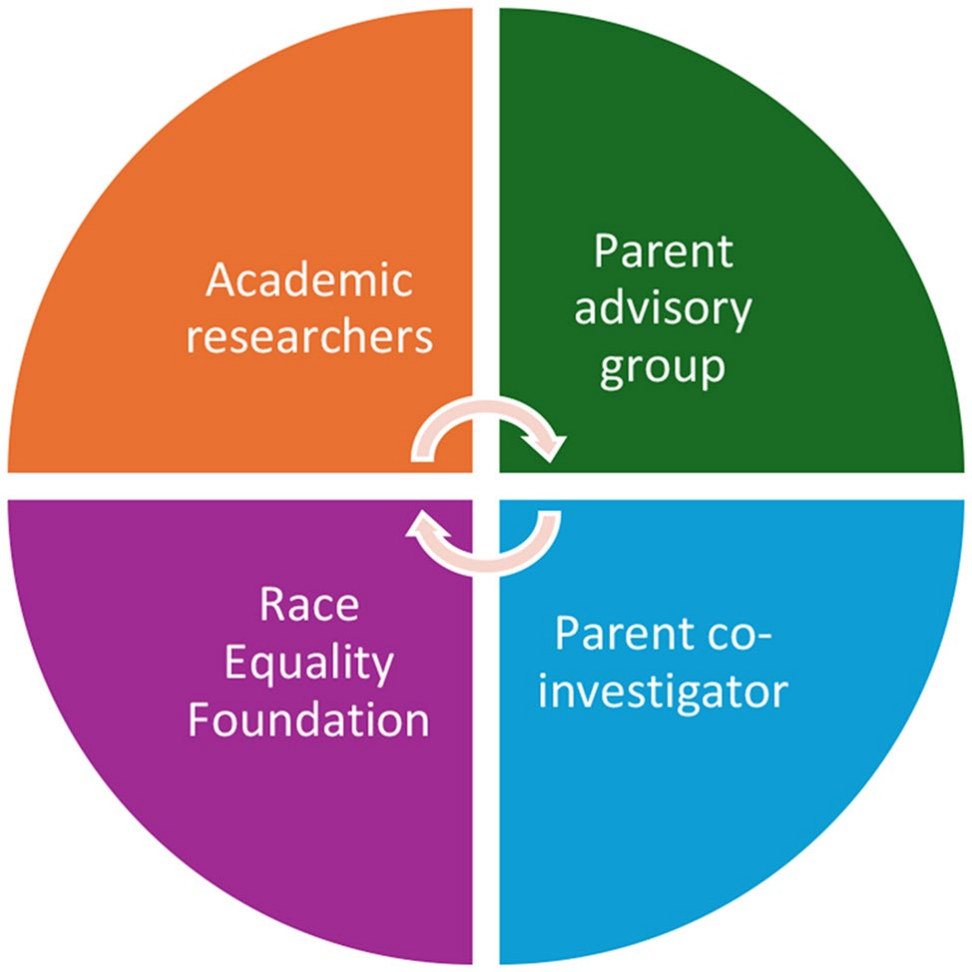
TOGETHER team partnerships

Led by Professor Richard Watt at UCL, TOGETHER was conducted across six urban sites in England—Calderdale, Hull, Kirklees, Leeds, London, and Luton—chosen for their social and ethnic diversity and alignment with real-world SFSC delivery contexts. The study launched in April 2019 and included an internal pilot, a full RCT, a cost-effectiveness evaluation, and an embedded process evaluation [27]. Recruitment was conducted through a mix of Local Authorities, community groups, and trusted local settings such as schools, children’s centres, and places of worship. A collaborative study team included academic researchers, a lived experience co-investigator, the Race Equality Foundation, and three Parent Advisory Groups (PAGs), composed of parents from participating communities (see Fig. 1).

The trial findings offer critical insights into how SFSC can reduce health inequalities, the study also sheds light on what it takes to conduct an inclusive, large-scale research project in practice. This paper focuses on the strategies and processes that enabled inclusive participation across a complex, multi-site trial.

## Aims and objectives

This paper offers critical reflections from the TOGETHER study and the key enablers to designing and delivering an inclusive research study in practice. Our specific objectives are to:

1. Explore participant recruitment and retention patterns and their links with specific inclusion strategies.
2. Examine the relational partnerships between academic researchers, the lived experience co-investigator, advisory groups, and the Race Equality Foundation.
3. Scrutinise the broader academic landscape in supporting or hindering strategies and practices for inclusive research.
4. Offer recommendations for researchers and funders to support equity and inclusion in research strategies and practices.

## Methods

### Study design

This paper presents a retrospective, critical reflective case analysis of the inclusive practices embedded within the TOGETHER study. The study began in early 2019 and during the study there had been interest to capture learning about the PPIE ‘work’ underway (i.e. with a regular survey with the advisory groups). The realities of delivering a complex trial meant that a formal evaluation was not planned but there was continued interest in drawing out insights, nonetheless. As such, this paper was developed following completion of the study as a critical reflective analysis of those involved, rather than a formal separate sub-study of the TOGETHER study. Rather than evaluating effectiveness through formal qualitative methods or fixed metrics, we sought to surface insights into the realities, and at times, the ‘messiness’ of conducting a large-scale community trial through our own reflections.

### Data sources

Our reflections were informed by multiple sources. First, we drew on descriptive trial data generated and overseen by the North Wales Organisation for Randomised Trials in Health and Social Care (NWORTH) Clinical Trials Unit (CTU), which held responsibility for trial data management and analysis. We relied on existing planned analyses of recruitment, withdrawal, attrition, and outcome data, and supplemented this with additional descriptive analyses relating to referral pathways into the study (i.e. from statutory services and community organisations) to uncover patterns of inclusion and engagement. We then reviewed five years of study team and PPIE meeting minutes. These documentary materials, originally generated for governance and operational purposes, were examined to identify how public involvement was discussed and enacted within study. We went on to share and discuss some of the insights from the trial data and documentary materials in two structured reflective sessions with the lived experience co-investigator and members of the PAGs. The sessions were documented and visually captured by a professional graphic illustrator (see.

**Fig. 2:**
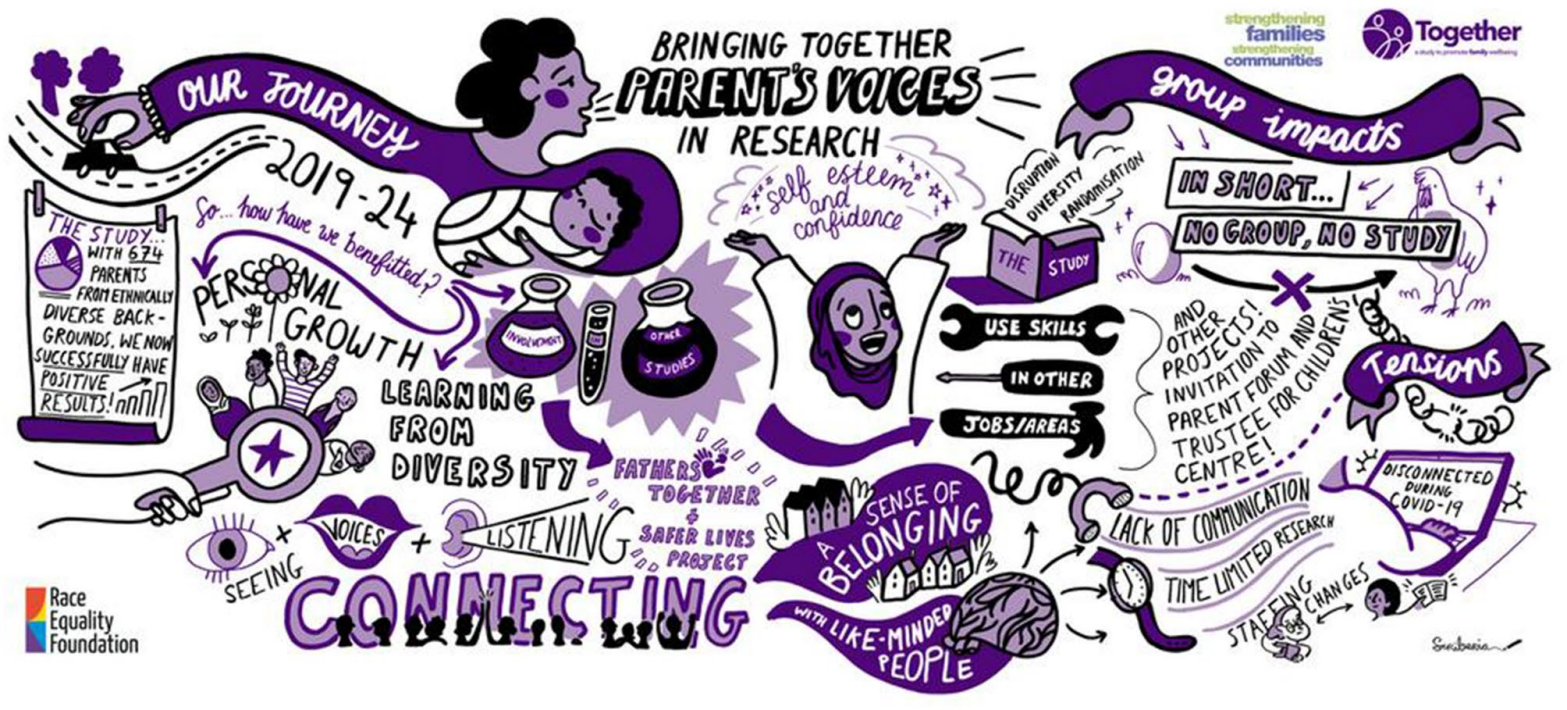
Parent advisory group visual reflections

Figure 2). In addition, some PAG members volunteered to participate in individual video interviews, which were compiled into a short film (see https://www.youtube.com/watch?v=x4cb71K9mUU) to support dissemination and knowledge mobilisation. These materials were intended to promote shared learning and visibility of the study rather than to serve as formal qualitative research data. All these forms of data were drawn on for the purposes of our critical reflections and findings presented in this paper.

### Analysis

The analytic process we followed was iterative, collaborative, and reflexive. AM first reviewed study documentation and descriptive trial data, including recruitment, retention, and referral patterns, to identify preliminary observations relating to inclusion and engagement across sites. These observations formed the basis for structured discussions with LB and KM, drawing on their sustained involvement in public involvement leadership and lived experience roles within the trial. These early conversations surfaced provisional themes relating to relational practice, infrastructure, leadership, and community partnership. These emerging insights were then taken into the first facilitated reflective session with PAG members in early 2024. The session was designed as a collective sense-making exercise, inviting participants to reflect on their experiences, perceived contributions, challenges, and impacts across the study journey. A second session focused on synthesising these reflections, identifying shared experiences, and articulating what PAG members considered to be important factors in supporting their meaningful inclusion and impact on the study. A professional visual scribe was engaged to document this process graphically to support collective reflection and knowledge sharing. Alongside this, PAG members, LB, KM, and AM discussed strategies for disseminating learning from the study. This included co-producing audiovisual outputs, such as a short film featuring consenting PAG members and key contributors. While primarily intended for dissemination and knowledge mobilisation, these materials also reinforced and clarified shared reflections about inclusion.

Following these sessions, AM, LB, and KM revisited all sources (documentary materials, descriptive data, reflective session outputs, and audiovisual transcripts) to identify recurring patterns which might be linked to equity, diversity, inclusion, and lived experience involvement and trial participation. Through iterative discussion and comparison across sources, five interrelated ‘enablers’ were identified as consistently underpinning inclusive participation and representation within the trial. A subsequent period of collaborative refinement involved additional researchers (AL, DC, and TBP) offering further reflections and an additional enabler. The study lead (RGW) and third sector co-investigator third sector lead (JB) also provided critical reflections as well as reflection on wider system-level factors that facilitated or constrained their enactment.

### Positionality and reflexivity

We acknowledge that our reflections are shaped by our positionalities, relationships, and proximity to the work on the TOGETHER study including our different roles, responsibilities, and forms of authority within it. The findings presented in this paper were primarily developed by three authors (AM, LB and KM), who worked closely together throughout different stages of the TOGETHER study and in the analysis presented here. AM is an academic researcher who held roles within the study; LB is Deputy CEO of the Race Equality Foundation and worked closely with delivery partners and community organisations; and KM is a parent co-investigator with lived experience who contributed to the study from its early stages through to dissemination. We connected through our respective roles in the study and developed an ongoing reflective dialogue about the PPIE processes and relationships that unfolded across the project. As women from minoritised ethnic backgrounds working across academic, third sector and lived-experience roles, we recognised both differences and commonalities in how we navigated the spaces surrounding the study. While our roles carried different forms of authority and power within the research process, we also shared experiences of working within racialised institutional contexts, including academic and research environments, which shaped how we understood issues of trust, representation and inclusion in the study. For LB and KM, reflections were also shaped by their positions as parents engaging with research concerning family life, while AM’s position as an academic researcher shaped how these insights were interpreted within broader research frameworks. Over time, including after AM had moved on from a formal role within the study, our continued collaboration across other research activities provided space to reflect more critically on these experiences. Towards the end of the study, we therefore sought to bring these reflections together in a more structured way as part of the analysis presented in this paper. While our own perspectives were an important starting point, we were also attentive to the limitations of relying solely on personal reflection. We therefore drew on additional sources including trial data, meeting minutes and reflections with the PAGs to situate, challenge and extend our interpretations. We also sought input from other members of the research team and study partners to sense-check the themes that emerged from these reflections. In particular, senior leads within the research (RW) and partner organisation (JB) contributed perspectives on how these insights resonated with broader context of research. Researchers involved in key roles on the study (AL, DC and TBP), including leading PPIE work during the study, also reviewed and reflected on the emerging themes to sense-check how these resonated with their own experiences of delivering and supporting the research. The findings in the paper therefore represent a situated interpretation shaped by our positions within and around the study rather than a neutral or exhaustive account of the PPIE processes. We therefore do not claim objectivity or completeness, but instead offer a situated perspective that embraces reflexivity, humility, and transparency. Our intention is not to present a definitive account, but to illuminate the key practices, tensions, and learning moments that supported inclusive research in this context. In the following section, we present key insights from these reflections, followed by a discussion around implications and recommendations for researchers and funders.

## Results

### Ethnically and socially diverse representation in the study

The TOGETHER study successfully recruited 674 participants and delivered 34 SFSC parenting programmes, meeting 100% of the target. The sample was both ethnically and socially diverse: 65% of participants identified as Black, Asian, mixed, or other minoritised ethnicities; nearly half reported a first language other than English; and more than half had household incomes under £20,000. 16% (*n* = 107) completed questionnaires in a language other than English. Retention was also strong: only 11% formally withdrew from the trial, with attrition rates of 28% at the post-intervention assessment and 30% at the six-month follow-up. There was no statistically significant difference in withdrawal or attrition rates by ethnicity, household income, age of child, site or language. Findings (due to be published) showed that SFSC significantly improved parental mental wellbeing and had positive effects on secondary outcomes including children’s socio-emotional wellbeing, parenting practices, and family relationships. These benefits were observed across ethnic and socioeconomic groups, suggesting the programme’s potential to reduce rather than reinforce inequalities.

### Key enablers for inclusivity

We identified six key enablers that were critical to the inclusive and successful delivery of the TOGETHER study. These reflect the relational, structural, and institutional factors that shaped how the research was conducted and who it reached. These key enablers include:

1. Leading with lived experience: Navigating power, emotion and endurance.
2. Disrupting from within: Parent Advisory Groups as agents of change.
3. Roots into the community: Building strong relational partnerships with local community organisations.
4. Speaking the same language: Community researchers supporting culturally and linguistically inclusive data collection.
5. Connecting the dots: The third sector as powering inclusion.
6. Investing in inclusion: Budgets, resources, and leadership.

#### Leading with lived experience: Navigating power, emotion and endurance

A core feature of the TOGETHER study was the appointment of a lived experience co-investigator, whose role spanned the full lifecycle of the research. Her contribution was pivotal—not only in supporting inclusive practices, but in bringing consistency, challenge, and lived insight to a complex trial. Having previously completed the SFSC programme herself, she offered a unique perspective that bridged participants and researchers. Her sustained involvement added depth and credibility to the research process, ensuring it remained grounded in the realities of the communities it aimed to serve.

The co-investigator was identified by the Race Equality Foundation during the early conception of the study. The team was keen to draw on a public contributor who had the lived experiences to contribute meaningfully to the study rather than draw on the ‘usual suspects’ [19]. She was not a ‘professionalised’ PPIE contributor although had some prior involvement on an advisory group for a smaller study. She brought lived experience as a Black parent raising a neurodiverse child. She is also neurodiverse herself.

Over the five-year duration of the study, she attended nearly all of the Trial Management Group (TMG) meetings, co-facilitated 25 PAG sessions, and took part in numerous planning and debriefing discussions with the research team. All of this was carried out alongside her professional work, voluntary responsibilities, and family life. The time commitment was significant and to support her role, the Race Equality Foundation offered consistent infrastructure and care. This included regular preparatory talks ahead of meetings and maintaining open lines of contact with other dedicated staff on the research team.

These relationships formed a support network that enabled her to stay engaged in what was often a demanding role. She had been instrumental in raising issues of power imbalance—reminding the team that the subject matter under study was not abstract or theoretical for the families involved. It was lived, personal, and at times painful. She reflected in an individual interview:

*This is not theory stuff for us…it affects my mental health… this is real for us.*

She frequently spoke to the discomfort of navigating academic spaces, where research language, professional hierarchies, and institutional norms could feel alienating. She continued where she reflected:

*I feel it’s not always been comfortable because sitting with researchers and different types of people around the table you actually question*,* can I say this? Can I be a part of this?*

There were moments where the emotional labour and role led her to consider stepping back. However, having a key contact point at the Race Equality Foundation was an important support function. This support included regular structured meetings, often prior to formal meetings as a space where she could ask questions, clarify terminology, and raise any emerging concerns in a supportive environment. These pre-meetings were not token gestures—they provided psychological safety and built confidence for full participation in formal research governance. There was also opportunity for informal and ad-hoc check-in’s which suited her schedule and needs. She reflects on this support:

*If it wasn’t for you guys at Race Equality [Foundation]*,* I would have walked a long time ago… This is not just research—it’s our lives.*

The lived experience co-investigator was a consistent presence over the duration of the study, which became particularly critical amidst multiple changes in the research team over the five years. This turnover and transitional periods between new research staff had some impact on continuity and relationships, especially with the PAGs, where trust and familiarity were central to sustained engagement. For PAG members, new research staff brought different levels of experience and varying commitment to PPIE. This required PAG members to rebuild trust with the research team and adapt to new relationships, approaches and sometimes extended gaps between meetings. The lived experience co-investigator became a stable and consistent anchor who understood the PAG’s perspectives and progress made with their involvement in the study and was able to advocate and offered reassurance. Her contributions left a lasting imprint on the study’s values, approach, and credibility, and she ultimately reflected:

*I think now looking at five years and my contribution … and seeing the end picture it’s like wow was I really a part of this? Did I actually make changes? And yeah I have*,* and it’s not just for me*,* I’ve done it for all parents.*

#### Public representatives as research partners: the role of Parent Advisory Groups

Active and sustained involvement of diverse parents as genuine research partners was a vital component of the TOGETHER study. While advisory groups are now a common feature in health research and trials, we looked to go beyond consultation and towards more embedded and ongoing partnership. The PAGs played a central role in shaping how the research was designed, delivered, and experienced by participants – often challenging assumptions and surfacing overlooked perspectives. We reflect on how the PAGs were initially established, how they helped shape and positively disrupt the study, and the ripple effects of personal growth and community impact.

#### Removing barriers to joining the advisory groups

From the outset, we sought to build advisory groups that reflected the diversity of the parents and communities we aimed to reach. PPIE groups can consist of individuals who are more socioeconomically privileged or already familiar with research, which limits their ability to speak to the experiences of families facing structural disadvantage, racism, or exclusion. To address this, we worked through trusted networks, particularly SFSC facilitators, Local Authorities, and community organisations, to invite parents who had completed the SFSC programme and reflected the social and ethnic backgrounds of our target participant population. To enable participation, we minimised common barriers by selecting accessible community venues and providing childcare, travel expenses, meals, vouchers, and interpretation support as standard practice. One parent noted:

*I’ve really enjoyed it. I’ve always tried to make time for these meetings and to participate as and when I can and the team have been very accommodating to me throughout the years in terms of childcare and transport and also bringing the newborn baby [to meetings].*

A dedicated Research Assistant based at the Race Equality Foundation also played a crucial role in building trust and maintaining relationships with PAG members. Having a key contact point provided a relational focus for the parents. In total, around 40 socially and ethnically diverse parents joined three PAGs (two in London, one in the North of England). They came from a range of ethnic and sociodemographic groups with notable representation of Black, Asian, and minority ethnic parents, single and partnered carers, and a range of family types. Over five years, a core group of around 24 consistently met around 25 times (14 in person, 11 online), with regular contact maintained via WhatsApp. This wraparound model fostered community, trust, and continuity.

#### Shaping the study through positive ‘disruption’

The PAGs influenced several key elements of the study’s design and conduct at an early stage of the study. During the pilot phase, for example, they raised concerns about the suitability of an outcome measure (parenting practices) selected by the researchers, noting that it focused too heavily on negative practices, offered little attention to positive ones, and was lengthy and difficult to complete. Their feedback prompted the research team to review alternatives with the PAG’s and replace the measure with one that struck a better balance between positive and negative practices and was more acceptable to complete.

PAG members also offered critical insight on randomisation and the use of a waitlist control—a concern raised by the research team and partner services. Contrary to assumptions, PAG members and the lived experience co-investigator accepted randomisation, noting that parents were accustomed to waiting for services and that randomisation introduced fairness and transparency in a process that usually felt opaque or arbitrary. They also accepted the rationale of randomisation in a robust evaluation and felt that other parents would too, if clearly explained. These discussions prompted the research team to shift how the study was presented: not solely as a route to receive an intervention, but as an opportunity for parents—particularly from underrepresented communities—to contribute to a research study.

These early discussions helped shape the trial’s internal pilot, where we were able to promptly enlist research sites, test our processes and establish if progressing to a full trial was feasible. We met 95% of our recruitment target and retained 89% of participants during the pilot phase which gave confidence to progress to a full trial [27]. In the lead up to a full trial, services still held some reservations around randomisation and the waitlist condition, so the research team also introduced quarterly study newsletters and delivered webinars as a way to discuss the research and encourage services to learn from each other’s experiences and hear from PAG members directly. The direct voices from the PAG continued to be a source of assurance and influence throughout the study in this way.

The research team had also planned to translate materials and run SFSC programmes in a range of community languages (as standard and advocated by the Race Equality Foundation). The PAGs supported and influenced this process by pushing for the inclusion of community researchers and champions. One PAG member shared how she took on a research ‘champion’ role in her area which helped address parents’ concerns about trust with the research. She reflects:

*I work in the community in a Nursery so we have lots of people coming and I have very good relationship with parents and when I asked them to be involved in this research some of them like said ‘yes’ and after said ‘no we don’t want to be part’. I said ‘why?’ and they said ‘maybe the information will go here and there’. I said ‘no the information will be only in the study no one will share your information’ and because they trust me they accept it and [agree] to be part of the research.*

Drawing on community members helped build trust in the research which likely boosted engagement and enhanced the quality and integrity of the data collected.

During the COVID-19 pandemic, the PAGs also played a vital role in helping the study rapidly adapt during a time of great uncertainty and concern. Many participants in the study at that point came from groups who were disproportionately affected by the pandemic and the PAGs urged the team to take an active role in supporting participants. They suggested that we could be a trusted source of information and support in our position as public health researchers with an existing link. The team co-developed a newsletter co-developed with the PAG which included clear, accessible public health messaging with practical tips on home-schooling etc. In total, 14 newsletters were shared with participants during 2020 as well as being shared more widely through other networks.

The PAG went on to advocate for a return to in-person formats for programme delivery, group meetings, and data collection—citing digital exclusion and the emotional toll of lockdowns on families as important considerations to maintain study recruitment and retention. For many research studies, progress stalled or paused and PPIE efforts with marginalised groups reduced with the transition to online methods [33]. In the TOGETHER study, this was a live and ongoing discussion and the PAG were clear in advocating for a return to in-person modes. This required negotiation with the research team who had adapted to remote methods and saw the value in offering a flexible mode of engaging whilst arguably protecting personal and public health with ongoing risks of COVID-19. A consensus was eventually reached with the return to in-person PAG meetings and SFSC programme delivery, but with online methods still maintained for data collection. These discussions and actions most likely helped maintain high levels of engagement with the PAG and maintain participant retention in study.

At times, PAG members also felt the impact of differing views within the research team about how PPIE should be enacted. While some researchers actively embraced the PAG’s input and emphasised PPIE, other researchers prioritised their involvement less. When combined with staff turnover, there were fluctuations where PAG meetings were regular with active involvement and other periods saw less frequent meetings which were largely consultative and focused on providing updates only. This inconsistency could be unsettling for parents who had invested in long-term relationships with the study. There were some missed opportunities for PAG members to contribute more consistently which impacted the quality of engagement.

Despite the strengths of the PAG model, certain limitations remained—particularly around the involvement of men and fathers. Just three fathers were active PAG members. While their contributions were thoughtful and consistent, the small number of male voices likely constrained the extent to which the study reflected fathers’ needs and experiences. This was reflected in the trial where only 5% of trial participants were male, despite SFSC’s usual reach of around 20% male participation [34]. As one father in the PAG reflected:

*I find sometimes researchers can be quite rigid with their task… we kind of create a bit of friction there and then that rejigs their thought process… I think it’s crucial that all research is done with lived experience parents.*

This concern was shared by the Race Equality Foundation, who regularly championed the need for a specific strategy to engage fathers (e.g. through dedicated fathers-only groups). It took time for specific action around the low uptake of fathers, and by the time several fathers-only groups were attempted the impact on overall father participation was limited. In hindsight, part of the challenge may have been shaped by the intense pressures of running a complex trial, with constant demands to meet recruitment and retention milestones. Within this context, the fact that all researchers on the ground were women may also have contributed to a blind spot around recognising the specific barriers fathers face. More broadly, however, the research was embedded within systems and services that are not father inclusive. Many of the community organisations we partnered with were staffed predominantly by women and were accustomed to working directly with mothers, who are often prioritised as the primary carers. Statutory agencies involved also had low levels of engagement with fathers, and the programmes allocated for the research were mainly scheduled during the day, making them less accessible to working men. These structural and systemic factors together constrained the representation of fathers in the study. Nonetheless, the PAGs reflected on their role on their involvement as concluded that: “*without this group there would be no study”.*

#### Ripple effects of personal growth and community impact

While the primary focus of the PAGs was to shape the study, members increasingly reflected on their own personal growth, skills, and confidence they gained through their involvement. These ripple effects—both individual and collective—highlight the wider value of inclusive research beyond study outcomes. From the outset, members were invited to share what they hoped to gain from their involvement and how they wanted to develop. Many set personal goals around confidence, public speaking, community leadership, and contributing to change. Over time, these aspirations were realised in a variety of ways.

Several PAG members reported taking on new community or professional roles directly inspired by their experience. One member reflected on how the PAG motivated her to become a Trustee of a local children’s centre, working with directors to improve facilities and activities for parents. A parent reflects:

*It’s encouraged me to continue being part of my community in many different ways and I’ve gone on to become a Trustee of the Children’s Centre*,* and work with the directors to improve the facilities and the activities that we have for the parents and in the community*,* and I do relate that back to this*,* you know being on this parent advisory group as well because it is very similar what we discuss and the format of the meetings.*

Another described how her first exposure to research through TOGETHER gave her the confidence to join a new NHS role as a patient safety partner and even consider studying research formally. She states:

*I never done any research [before] but to be part of this research was a very big thing for me and it give me like push to be part of different research*,* like I recently involved in a job with the NHS as a patient safety partner and I was using some experience from this research to do my other things…. I think like maybe I can go and study to do some research*,* yeah I find it very very helpful*,* great.*

In some cases, references were provided by the research team to support PAG members gain these opportunities. Other examples included members writing newsletter articles, speaking at webinars, contributing to national policy discussions, and representing the group in external forums. One participant went on to train to deliver the parenting programme in her area (outside of the research), while another established a parent-and-baby drop-in space in her community.

These reflections show that advisory group involvement can be mutually beneficial through enhancing the quality, relevance, and legitimacy of research, while also supporting confidence, voice, and leadership within communities. In this way, inclusive research practice does not only shape knowledge but can actively build social capital and capacity in those who participate. Further reflections and insights directly from PAG members can also be found here: https://www.youtube.com/watch?v=x4cb71K9mUU.

#### Roots into the community: Building deep, relational partnerships with local community organisations

Our third enabler centres on the importance of intentional recruitment strategies and relational partnerships to ensure inclusive participation, particularly among ethnically and socially diverse families.

#### Working within and beyond statutory agencies

In the TOGETHER study, we sought to reflect the open-access ethos of the SFSC parenting programme, which is grounded in prevention and universal access, not crisis-driven or a narrowly targeted intervention. We deliberately kept recruitment pathways broad and accessible, accepting both self-referring parents and those referred through a variety of services. Participants entered the study through Local Authorities, community organisations, family support services, schools, social work and criminal justice professionals, and community-based outreach activities like coffee mornings. Parents could also self-refer based on local promotion or word of mouth. This approach aligned with SFSC’s ethos that parenting support should be proactive and preventative, not restricted to families already in crisis.

Adopting this approach required careful negotiation with delivery partners. Statutory services, often operating under constrained budgets, tend to prioritise families with the most acute or immediate needs. While understandable, this form of gatekeeping can inadvertently reinforce inequality—limiting access for families who do not meet narrow thresholds, but who would still benefit from support. Similar concerns are often raised in research, where open-access recruitment is sometimes seen as a risk to methodological integrity, fearing it may ‘dilute’ the sample by attracting more resourced or ‘worried well’ parents.

We were keen to consider the balance between universal access, inclusion and equity. We adopted a version of ‘proportionate universalism’ where our strategy was universal but targeted proportionate to the level of disadvantage and actively addressing the greater barriers faced by some [1]. We focused efforts on areas with high levels of deprivation and ethnic and social diversity but recognised the barriers in accessing statutory agencies. We therefore partnered with 16 agencies and delivered 34 SFSC programmes: 19 led by Local Authorities (56%) and 15 led by community organisations (44%) (see Table 1). Together, they contributed to reaching, recruiting, and retaining an ethnically and socially diverse sample. Community organisations recruited 45% of participants and Local Authority led sites recruited 55%. Self-referral was higher in community organisation sites, as expected given their community-based nature.

**Table 1:** Recruitment and programmes by referral agency type

	Community organisations		Local Authorities		Total
Number of SFSC programmes	15	44.1%	19	55.9%	34
Number of participants consented/baselined	302	44.8%	372	55.2%	674
Average number of participants per programmes*	20		20		20
Participant self-referred	294	97.4%	305	82%	599
Participant referred	8	2.6%	67	18%	75
Number of participant withdrawals	34	44.2%	43	55.8%	77

* includes SFSC group and waitlist group

Each pathway brought different strengths and, in combination, helped ensure inclusivity (see Table 2). Examining demographic characteristics collected from baseline interviews, we found some variation in the participants recruited through Local Authority or Community Organisation sites. Local Authority sites had more participants reflecting single parents or those not in a relationship, those who are unemployed, and with a split between White British and racially minoritised groups. In contrast, community organisation sites brought a greater proportion of racially minoritised ethnic groups and participants who spoke multiple languages or from migrant backgrounds. Furthermore, there was a greater proportion of participants living in council housing and on low incomes and being housewives/husbands rather than unemployed or employed. Although the overall proportion of fathers and men was low in the study, those that were in the study largely came through statutory referral routes.

**Table 2:** Participant demographics by referral agency type

	Community organisation	Local Authority
Gender of parent		
Female	11 (3.6%)	22 (5.9%)
Male	291 (96.4%)	350 (94.1%)
**Relationship with Child**		
Birth mother	286 (94.7%)	339 (91.1%)
Birth father	6 (2%)	20 (5.4%)
Other (incl. adoptive, foster or stepparent)	10 (3.3%)	13 (3.5%)
**Parenting structure**		
Two-parent family	209 (69.2%)	176 (47.3%)
Single parent	85 (28.1%)	178 (47.8%)
Other (incl. step, foster, grandparent)	8 (2.9%)	18 (4.8%)
**Ethnicity of parent**		
White British	11 (3.6%)	187 (50.3%)
Black, Asian and minoritised groups	291 (95.4%)	185 (49.4%)
N/A	3 (1%)	1 (0.3%)
**Ethnicity of Child**		
White British	12 (4%)	184 (49%)
Black, Asian and minoritised groups	290 (95%)	188 (50%)
N/A	3 (1%)	1 (0.3%)
**Religion**		
Muslim	233 (77.2%)	79 (21.2%)
No religion	23 (7.6%)	150 (40.3%)
Christian	36 (11.9%)	121 (32.5%)
Other (incl. Hindu, Sikh, Buddhist and others)	10 (3.2%)	22 (5.8%)
**First Language English**		
No	234 (77.5%)	98 (26.3%)
Yes	67 (22.2%)	273 (73.4%)
NA	1 (0.3%)	1 (0.3%)
**Born**		
Abroad	251 (83.1%)	126 (33.9%)
UK	51 (16.9%)	245 (65.9%)
N/A		1 (0.3%)
**Highest Education**		
College degree or higher or NVQ Level 4–5	172 (57%)	189 (50.8%)
Secondary school (18 years) or NVQ Level 1–3	78 (25.8%)	116 (31.2%)
Secondary school (16 years)	35 (11.6%)	59 (15.9%)
Primary school or none	17 (5.6%)	7 (1.8%)
NA	0 (0%)	1 (0.3%)
**Housing Type**		
Owner occupier	21 (7%)	97 (26.1%)
Council rented	133 (44%)	100 (26.9%)
Housing Association	62 (20.5%)	54 (14.5%)
Private rented	70 (23.2%)	91 (24.5%)
Other	16 (5.3%)	27 (8.2%)
NA	0 (0%)	3 (0.8%)
**Household Income**		
Under £9,999	70 (23.2%)	94 (25.3%)
£10,000 - £19,999	98 (32.5%)	88 (23.7%)
£20,000 - £29,999	36 (11.9%)	33 (8.9%)
£30,000 - £39,999	13 (4.3%)	24 (6.5%)
£40,000 - £49,999	9 (3%)	17 (4.6%)
Over £50,000	13 (4.3%)	37 (9.9%)
NA	63 (20.9%)	79 (21.2%)
**Employment Status**		
Employed	66 (21.9%)	115 (30.9%)
Student	23 (7.6%)	11 (3%)
Housewife/husband	130 (43%)	81 (21.8%)
Unemployed	67 (22.2%)	132 (35.5%)
Other (incl. retired)	14 (4.6%)	33 (8.9%)
NA	2 (0.7%)	0 (0%)

Importantly, baseline needs such as mental wellbeing, child wellbeing, parent-child relationships, parenting practices, self-efficacy, and neighbourhood and community cohesion did not differ by community organisations or Local Authorities sites (see Table 3). Therefore, dispelling concerns regarding ‘diluting’ need when recruiting outside formal statutory services. This highlights the strength of a mixed recruitment model—ensuring inclusion without compromising comparability.

**Table 3:** Participant baseline measures by referral agency type

Outcomes (measure)	LA or CO	*N*	Mean	% Missing
Parental mental wellbeing	LA	364	46.45	2.2
	CO	296	52.21	2
Parental self-efficacy (PMS)	LA	356	19.16	4.3
	CO	293	18.96	3
Child socioemotional well-being (SDQ Total Difficulties Score)	LA	368	17.31	1.1
	CO	302	13.14	0
Child socioemotional well-being (SDQ Impact Score)	LA	370	2.84	0.5
	CO	302	1.43	0
Positive parenting practices (MAPS)	LA	371	4.42	0.3
	CO	300	4.39	0.7
Negative parenting practices (MAPS)	LA	370	1.95	0.5
	CO	300	1.93	0.7
Parent-child relationships - conflict (CPRS)	LA	361	23.63	3
	CO	295	19.87	2.3
Parent-child relationships - closeness (CPRS)	LA	356	30.71	4.3
	CO	298	31.48	1.3
Quality of partner relationships (QMI)	LA	197	35.62	0.8
	CO	209	37.62	0.7
Neighbourhood cohesion –(adapted Buckner Scale)	LA	367	24.57	1.3
	CO	299	26.28	1
Social cohesion –(adapted Buckner Scale)	LA	370	28.18	0.5
	CO	296	29.2	2
	CO	286	73.96	5.3

#### Embedding cultural and language inclusivity

The TOGETHER sample included 65% of participants identifying as Black, Asian, mixed, or other minoritised ethnicities; almost half reporting a first language other than English; and more than half living in households with incomes under £20,000. Community organisations were particularly effective in engaging families who may be reluctant to access statutory services. The community organisations brought agility, cultural competence, and deep community trust. Many had longstanding relationships with the Race Equality Foundation, and recruitment efforts built on these existing links. Organisations such as Westway Trust, Minik Kardes, and the Somali Youth Development Resource Centre played vital roles in reaching Arabic, Turkish, and Somali speaking families. Their involvement enabled the study to run targeted, community responsive SFSC programmes—for example, a Somali speaking group for Somali women in Camden, and a mixed Arabic/English group at a youth arts centre in North Westminster.

One notable example was the partnership with the London-based Muslim Community Association (MCA) in Tower Hamlets (https://mcasite.org/). The study originally aimed to partner with the Local Authority, but despite a long-standing relationship with several staff and multiple conversations, staffing shortages and challenges with the parenting service in the borough made this unfeasible. The Race Equality Foundation were clear that the Bangla-speaking community in East London were an important ethnic group with high take up of SFSC and should not be missed from the study. As a result, they facilitated a collaboration with MCA and a local SFSC facilitator, resulting in the successful delivery of three Bangla-speaking SFSC programmes (plus three more for waitlist participants after the study).

Crucial to the success of this collaboration was both the local SFSC facilitator, who had delivered the programme for more than a decade in the borough and her reputation as someone imbued with expertise and trust, but also the support provided by MCA. Several key people within MCA and East London Mosque had trained as SFSC facilitators and the programme had been previously delivered to mothers and fathers through the organisation. This meant both the Race Equality Foundation and the programme were trusted entities. This existing relationship was key in allowing for rapid mobilisation and the roll out of the research.

Sessions were hosted in known venues such as a Muslim Women’s Centre and a nursery at The London Muslim Centre attached to East London Mosque, with local and trusted Bangla-speaking facilitators and childcare providers. Participants were recruited through mosque announcements and other staff and volunteer communications, word-of-mouth, drop ins and peer networks. Facilitators worked closely with the research team to ensure ethical consent, clear communication, and culturally appropriate data collection.

Translation was another area where thoughtful planning, adequate budget, and infrastructure support were critical. In areas with a high proportion of families from ethnic minority groups, Local Authorities and community organisations often run SFSC groups in languages other than English (most commonly Turkish, Somali, Bengali, and Arabic). To enable families who required language support to fully participate in the study, all core materials, including consent forms, participant information sheets, adverts, and letters, were translated into key community languages.

The study followed a rigorous, WHO-informed translation process to ensure materials were not only accurate but also culturally and contextually appropriate [35]. Translations were reviewed by bilingual community members, some of whom were Parent Advisory Group members, alongside researchers to ensure clarity, resonance, and relevance. Back-translations were used to check conceptual alignment with the original text, and final versions were tested with target community members. This collaborative approach took time, care, and financial investment which ensured that translated materials were meaningful, accessible, and respectful to the families involved.

This approach reflected a wider ethos: inclusion cannot be ‘bought in’ through transactional services. It must be embedded through relational, context-sensitive practice underpinned by trust, understanding, and a deep commitment to removing barriers to participation. Community organisations played a central role, not just in reaching families, but in co-shaping how the study was delivered in different local contexts.

#### Building partnerships – not extracting resources

Crucially, local community organisations were not treated as simply delivery channels. They co-produced engagement strategies and ensured sessions were delivered in known venues, supported by multilingual staff, and aligned with participants’ ethnic, linguistic and cultural needs. The research team co-hosted a range of events to reach and recruit parents including, coffee mornings, sharing information via mosque newsletters and Friday announcements, organising online Q&A sessions and in-person info events, and mobilising SFSC-trained community facilitators to spread the word through everyday interactions.

Another example of this approach was our partnership with the Westway Trust (https://www.westway.org/), a London-based organisation in North Kensington’s diverse local community. Westway’s team both reflected and represented the community and had long-standing connections through a network of over 60 member organisations ranging from residents’ associations and supplementary schools to sports clubs and local charities. We approached them during the early pilot phase knowing that we were still in the learning phase of recruitment and needing partners willing to learn with us. Early discussions covered the research need with openness around the partnership for mutual learning (rather than immediate requests or expectations). We started conversations with discussions around what programmes they wished to run, what strategies might work to engage the community, any training and resource needs, and what they might gain from the partnership. Westway staff were enthusiastic, seeing the research as an opportunity for local parents to attend the programme and for the organisation to further support community wellbeing.

The Westway Trust brought invaluable insights into how best to engage parents and connecting the researchers to their existing networks and communities. Researchers joined Westway staff at various events, many in the evenings and weekends to visit supplementary schools, meet families, and hand out materials. During particularly intensive periods of the study, the research team became more embedded with the Trust with dedicated space for working and collaborating together. These efforts that not only boosted recruitment but also built trust and legitimacy for the research with the visible collaboration with a trusted organisation. They also brought ideas on where to run the SFSC parenting programmes and provision of appropriate food and childcare facilitates (i.e. some programmes were run in a private room in a soft play centre which had a large window for parents to see their child under the supervision of quality childcare). Lessons learned from Westway helped shape our study processes across all our sites and the collaboration continued beyond the pilot phase where they continued to run programmes and recruit parents as well as support translate of research materials. Westway went on to be one of our most successful research sites, delivering five of the 34 SFSC parenting programmes in the trial and bringing in the second-highest number of participants. This close working relationship was both productive and personally rewarding for the research team.

What made these partnerships work was mutual respect and reciprocity. Local community organisations are sometimes seen as mere ‘referral providers’ and some organisations express frustration where they are contacted by with requests to ‘pass on’ ethnically and socially diverse communities they have spent time and resources to cultivate. These requests can feel extractive with little discussion, interest or long-term investment in their work. In contrast, our approach sought to build relationships that were mutually beneficial with discussions around any training needs, cost and resources needs and how we could support and promote their work (e.g. through branding and public recognition of shared successes). This helped shift the dynamic from extraction to partnership.

While statutory services played a vital role, particularly in reaching families with high or complex needs, the involvement of local community partners ensured greater diversity, stronger trust, and more culturally appropriate delivery.

#### Speaking the same language: Community researchers supporting culturally and linguistically inclusive data collection

Building on these community partnerships, our fourth enabler focused on the role of community-embedded researchers, who were critical for recruiting and collecting data from culturally and linguistically diverse groups. While community organisations opened the door to participation, it was the community researchers, who sustained participant engagement throughout the study. Using the translated materials developed in collaboration with community organisations, they conducted recruitment interviews as well as follow-up data collection interviews in participants’ first languages, to provide clear understanding, build trust, and cultural resonance.

Seven community researchers were trained and supported over the course of the study. They reflected the linguistic and cultural diversity of participating families, collectively speaking Arabic (*n* = 3), Somali (*n* = 1), Turkish (*n* = 2), and Bengali (*n* = 1). Two community researchers had a background in psychology, one held a PhD, one was a dental public health student, and three had no formal research experience. Training covered the research protocol, randomisation procedures, ethical and consent processes, and data collection methods. For many, this was their first formal role in research, and the research team invested significant time and care to train, support and build confidence whilst ensuring fidelity to study procedures. Ongoing supervision, regular check-ins, and peer support were crucial to sustaining engagement, particularly as community researchers were often balancing caring responsibilities, employment, study, and community commitments alongside the research role.

The community researchers understanding and closeness within their communities was both a strength and a challenge. On one hand, it enhanced trust, facilitated informed consent, and allowed nuanced communication about a complex trial. Community researchers could explain randomisation in culturally and linguistically appropriate ways, using metaphors and everyday examples that resonated with participants. On the other hand, their closeness to participants occasionally blurred professional boundaries, creating emotional and ethical tensions that required careful support. For example, researchers were intended to remain ‘blind’ to participants’ allocation, but this was sometimes compromised, given how embedded the community researchers were within their local networks.

The team addressed these challenges through flexible supervision, reflective discussions, and ongoing support. The community researchers were invited to our weekly data collection meetings. These meetings were for researchers involved in data collection and created a space to reflect on their week and openly discuss any challenges encountered. These meetings were used to reinforce principles such as maintaining confidentiality, avoiding conflicts of interest, and managing expectations about the research, which was particularly important since community researchers sometimes held personal connections with potential participants which required ongoing training and discussions with the wider team. These experiences suggests that research teams working with community researchers should anticipate that boundary challenges may arise particularly when researchers are recruited because of their strong community connections. Regular reflective supervision, opportunities to discuss dilemmas collectively, and clear guidance to support genuine embedded community-based research.

Variations in community researchers’ confidence, availability, and research experience also created unpredictability and could be demanding for the research team at times. Practical issues, such as delays in reimbursements for the community researchers or complications relating to benefits and carer allowances, added additional strain. Dependence on individual researchers also posed challenges. For instance, for some SFSC language groups there was only one community researcher available for follow-up interviews, so the study relied heavily on them. Identifying and training new community researchers was time consuming for the research team with data collection windows being just four weeks. Balancing relationships, supervision, training, and management of the community researchers required careful coordination and flexibility from the research team.

Yet despite these challenges, the community researchers were indispensable, particularly for follow-up data collection, where their local knowledge and persistence helped achieve the high retention rates. Their contributions went beyond operational delivery; they acted as cultural interpreters and advocates, helping the team navigate community norms, sensitivities, and expectations. By involving community members as researchers, the TOGETHER study exemplified inclusion not only in who participated but also in who conducted the research. At the same time, it exposed the limitations of traditional research infrastructures which are often ill-equipped to support community-based employment, flexible payment, or training pathways within the required timelines.

#### Connecting the dots: The third sector as powering inclusion

Our fifth enabler centres on the pivotal role of the third sector, specifically, the Race Equality Foundation, in ‘connecting the dots’ between lived experience, community engagement, and academic research. They played a central role in ensuring that the TOGETHER study was inclusive, prioritised lived experience, and meaningfully connected to the communities it sought to engage.

From the outset, the overall research leadership was intentional about having a lived experience co-investigator and creating an open, accessible space where all forms of expertise could be shared and valued equally. This clarity of vision set the tone for collaboration. The academic lead brought deep experience of RCT’s, methodological rigour, and academic credibility; the Race Equality Foundation contributed its expertise in the SFSC programme and community engagement; and the lived experience co-investigator brought first-hand knowledge of the realities facing parents. Recognising and valuing these distinct contributions early established mutual respect, trust, and a shared sense of purpose and enabled the Race Equality Foundation to fully engage and offer expertise. This commitment was backed by dedicated research resources and funding.

As co-investigators and members of the core research team (see Fig. 1), the Race Equality Foundation brought a unique combination of content expertise in the SFSC programme, longstanding relationships with delivery partners, and extensive understanding of anti-racist practice. Their role was not peripheral or symbolic—they ‘powered inclusion’ by acting as advocates, translators, connectors, and critical friends throughout the study’s lifecycle. For community organisations and delivery partners, the Foundation advocated for the time, training, and resources required to implement SFSC in the context of a complex research trial.

For the lived experience co-investigator, the Race Equality Foundation created a space of psychological safety and support, holding regular pre-meetings ahead of formal governance structures to explain terms, clarify expectations, and process concerns. This consistent scaffolding enabled her full and confident participation in decision-making and helped maintain her engagement across a demanding study. For PAGs, the Foundation provided critical infrastructure by coordinating meetings, handling expenses, maintaining regular contact, and ensuring their input remained visible and valued even during periods of heightened pressure around recruitment or data collection. In many ways, the Race Equality Foundation acted as the study’s ‘connective tissue’ in being able to identify barriers to inclusion and guiding the process so that community voice, lived experience, and structural equity were embedded at the heart of its design and delivery, not treated as add-ons. For instance, when community organisations were invited to deliver programmes and support recruitment, the Race Equality Foundation helped guide these partnerships to be meaningful and not extractive through ensuring costs were covered and providing ideas for outreach activities, refreshments and childcare provision, community-language facilitators from their past experience working within communities. More broadly, the Race Equality Foundation also helped sustain a relational approach to involvement across the study during intensive phases of the research where engagement with the PAG reduced with the demands of delivering other aspects of the study. Here, the Race Equality Foundation encouraged sessions which engaged the PAG’s in reviewing emerging findings and supporting sense making (particularly relating to the process evaluation).

Working across academic, community, and lived-experience domains brought richness but also complexity, requiring ongoing translation, negotiation, and trust-building. In this way, the third sector did more than enable participation, it powered inclusion. It helped to reframe whose knowledge counts, how research relationships are formed, and what it means to centre equity in a large-scale trial.

#### Investing in inclusion: Budgets, resources, and leadership

While inclusion is often talked about in terms of values and intent, delivering inclusive research required concrete investments of time, money, infrastructure, and leadership. The success of the TOGETHER study was not only the result of community partnerships or public involvement, but also of the structural conditions that supported them. This final enabler underpinned and enabled all the others.

Dedicated budgets for involvement and delivery were essential. From the outset, the study team recognised the importance of fairly resourcing those involved, including public contributors and third sector partners. The PPIE lead role was properly resourced with payment, travel and subsistence costs covered making sustained involvement possible over the five-year study. The Race Equality Foundation managed logistics and payments for PAG members, allowing reimbursements to be made promptly and with less bureaucracy than university systems typically allow. Resources were also allocated to ensure inclusive PAG meetings, with childcare, meals, interpreters, travel arrangements, and digital support made available to reduce barriers to participation.

Infrastructure support was important, but this was not always consistent. The Clinical Research Network (CRN) (now called Research Delivery Network [RDN] since the research concluded) provided important infrastructure support to the trial. In some CRN localities, individuals within these systems played a critical role in championing inclusive approaches by offering staff, budgets and support whilst removing barriers for the research team to access this support. For example, three CRN researchers supported the data collection team with conducting interviews which was invaluable during busy times and allowed us to comfortably reach participant targets in those areas. However, this was not universal; some CRNs were far more engaged and helpful than others. Furthermore, there were limits to how much CRN researchers could support aspects of the study such as building partnerships with community organisations and the PAGs. CRN staff were often working across several projects rather than providing dedicated, sustained time to the study, which made it difficult to develop the kinds of relationships that were essential for inclusive research. This inconsistency highlights the importance of not just having infrastructure in place, but ensuring the right people are in key roles, people who value inclusion and are willing to put in the work to make it happen.

The study benefited from leadership that was not only open and values-driven but also practically engaged. The Chief Investigator was visible, approachable, and responsive, playing a key role in creating a culture of respect, inclusion, and mutual learning. The academic team remained open to challenge from the PPIE lead, Race Equality Foundation and community partners, even when this was uncomfortable or required rethinking previously agreed plans. Inclusion was not a side activity it was woven into the study’s core, supported by people, processes, and infrastructure.

Yet the reliance on individual staff members to champion inclusion also created vulnerability. When those individuals moved on, the emphasis on inclusive practice sometimes waned until others took up the mantle. This highlighted how inclusion can hinge on personal commitment rather than being embedded as a non-negotiable aspect of research infrastructure. These vulnerabilities underline a wider truth: without the time, funding, care, and leadership to support this work, even well-intentioned inclusion efforts can quickly become tokenistic or unsustainable. At the same time, the TOGETHER study showed that when these conditions were in place, even within a challenging academic environment, meaningful inclusion could be achieved at scale, leaving a legacy that influenced both the research team and participating communities.

## Discussion

The TOGETHER study set out to evaluate the effectiveness of the SFSC parenting programme for ethnically and socially diverse families in England. While the primary trial findings will be reported elsewhere, this paper has focused on the underlying strategies that supported inclusive research practice—particularly the partnerships and processes that enabled recruitment, engagement, and retention of a diverse sample. Six key enablers were identified: (1) lived experience leadership through a co-investigator; (2) public involvement via local Parent Advisory Groups; (3) relational partnerships with community organisations; (4) community researchers (5) strategic support from a third sector organisation, the Race Equality Foundation, and (6) investing in inclusion through dedicated budgets, resources, and visible, supportive leadership.

These findings add depth to current agendas around inclusive research and PPIE by providing practical insight into how inclusion can be embedded into complex trials. These findings resonate with critiques of tokenistic or performative approaches to PPIE, where public involvement is reduced to procedural compliance rather than meaningful influence [22, 23, 25]. In contrast, our case illustrates how relational infrastructure and sustained partnership can shift PPIE from symbolic inclusion toward something more transformative which supports the representation of ethnically and socially diverse groups in research. Health research has long failed to adequately represent ethnically and socially diverse communities [9, 10]—raising both ethical and scientific concerns [7, 8, 36]. Our findings highlight that inclusive and equitable research is fundamentally relational, built on trust, cultural understanding, and shared ownership with communities. These relationships cannot be engineered solely through policy agendas, tools and checklists alone; they must be cultivated through sustained engagement and a willingness to confront power imbalances. Our approach to public involvement developed gradually - rooted in transparency, humility, and a readiness to listen, learn and adapt. This process was not always smooth. It required navigating disagreement, discomfort, and the constraints of a research system often ill-suited to relational practice. Yet it was precisely this openness to challenge that strengthened the study’s integrity and relevance. Importantly, the work of the PAGs extended beyond shaping study procedures. During the COVID-19 pandemic, PAG members encouraged the research team to recognise its position as a trusted actor within participants’ lives, prompting the co-development of accessible public health newsletters and practical support. Similarly, the study created opportunities for PAG members’ own leadership and development beyond the confines of the trial. These experiences highlight that inclusive research does not occur outside the social world of participants; rather, it situates researchers within that world, implicating them not only as observers but as relational actors with responsibilities that extend beyond data collection. The study offers a compelling example of how PPIE and EDI, when properly aligned, can move beyond tokenism to drive genuine transformative change.

A core insight from this study is that inclusion cannot be achieved through goodwill alone. It takes investment of time, money, and people. Inclusive research demands space to build relationships, flexibility to adapt to community needs, and leadership that values process as much as outcomes. Dedicated capacity such as embedded roles within the Race Equality Foundation and funding for PAGs was not a nice-to-have, but essential. Without this, inclusive intentions risk becoming empty rhetoric. Leadership also mattered. Inclusion was supported because the Chief Investigator, key members of the research team and partners actively prioritised it not just in principle, but in the decisions that shaped staffing, governance, and resourcing.

The findings also have implications for how research proposals and interventions are assessed for ‘value for money’. Inclusive research requires dedicated staffing, community partnerships, supervision structures, and sustained engagement — all of which carry real costs. If funding boards and commissioners fail to recognise and resource these elements explicitly, inclusion risks being deprioritised in favour of cheaper, faster, but less equitable approaches. In the TOGETHER study, our inclusive approach was vital to producing robust and reliable research. Value for money assessments must therefore account not only for intervention delivery costs, but for the relational and infrastructural work required to ensure equitable participation and impact.

Another central learning from TOGETHER is that inclusive and equitable research requires rethinking not only methods and approaches, but the academic systems that underpin them. Our findings raise fundamental questions about whether the current research environment is equipped or even designed to support the kind of relational, flexible, and community-rooted work that inclusion demands. Over five years, the study experienced multiple changes in core research staff, whilst there was relative stability across the Race Equality Foundation and PAGs. For PAG members, this contrast created a clear tension: while their own commitment to the study spanned several years, they often had to reintroduce themselves, revisit previous contributions, and adapt as new researchers brought different levels of experience and varying priorities around PPIE. These experiences underline how precarious employment structures in academia can undermine continuity in inclusive research, with real consequences for trust and long-term community relationships.

These staffing transitions also speak to wider systemic issues. Researchers, especially those early in their careers, operate in a context shaped by intense pressure to publish, secure funding, demonstrate ‘impact’, teach, and deliver high-quality research, all within tight timelines [37–39]. Within this system, PPIE is often valued in principle but undervalued in practice and positioned as peripheral to the outputs that drive academic success. It does not easily fit within traditional indicators of performance, such as publication metrics or grant income, and it is rarely resourced or supported as a core part of research infrastructure. This disconnect can be surprising to those outside academia who may assume that universities offer stable platforms for community partnerships. In reality, many academics experience considerable precarity and must juggle competing demands without institutional incentives for inclusive practice. As a result, inclusive PPIE efforts, no matter how well-intentioned, risk being fragmented or deprioritised. Inclusion becomes aspirational rather than operational. However, several researchers who worked on TOGETHER have since moved into roles that prioritise PPIE or meaningful participation, showing how the study’s influence extended to shaping individual career paths, even if systemic conditions remain fragile.

Against this backdrop, the Race Equality Foundation’s involvement became a stabilising force in ensuring continuity, cultural competence, and ethical integrity across all stages of the trial. This highlights the critical but often under-resourced role of third-sector organisations as cultural brokers and custodians of community trust within health research [26]. What made this partnership work was not just the Race Equality Foundation presence, but how the relationship was structured. They were involved from the outset as a co-investigator, not added once the protocol was finalised. Their expertise was treated as integral, not supplementary. However, it is vital not to idealise this model. The Race Equality Foundation itself operates in a precarious funding environment, reliant on external grants and project-based income. In addition, not all third sector or community organisations have the expertise, confidence, capacity, or infrastructure to engage with complex research processes in this way. To build on what TOGETHER achieved, there must be real investment in third sector capacity, not just short-term subcontracting, but long-term partnerships based on mutual respect and shared power.

This paper offers reflections and insights on the issues of inclusion in health research, but we recognise that these are inherently partial, shaped by the perspectives of the authors, and may carry blind spots, omissions, or unacknowledged biases. We do not seek to reflect every aspect or experience on the TOGETHER study, nor did we seek to comprehensively scrutinise every enabler, challenge, or structural factor influencing how equity and inclusion played out within the study. Nonetheless, we hope that sharing this reflective and sometimes messy learning helps to open new conversations and invite further dialogue, critique, and exploration of how inclusive research can be better understood, practiced, and supported in real-world contexts. The broader implication is clear: for inclusive research to become standard practice, not the exception, we must shift the structural foundations of how research is funded, governed, and evaluated. To move from ‘tokenism to transformation’, we offer four recommendations for researchers, funders, and institutions:

- Funding long-term partnerships, not just project-based collaborations.
- Recognising third sector expertise and organisation as critical research partners.
- Reconfiguring academic reward systems to value engagement, relationship-building, and inclusion—not only outputs.
- Ensuring equity in power, resources, and voice across all partners, including community representatives and those with lived experience.

The TOGETHER study demonstrates that inclusive research is possible, but it takes time, budget, resources, and intent and commitment. Without structural change in how research is funded, staffed, and evaluated, such inclusion will remain the exception, not the rule. We cannot simply talk about equity, we must build the conditions that allow it to thrive.

## Conclusion

The TOGETHER study demonstrates that inclusive research within large-scale trials is achievable but requires deliberate choices and sustained work. Recruiting and retaining an ethnically and socially diverse sample required more than translated materials or recruitment targets but also depended on continuity of relationships, practical support for participation and partners who could work across academic and community contexts. Across six interlinked enablers, including lived experience leadership, active PAGs, deep community partnerships, multilingual community researchers, third-sector partnership, and dedicated resourcing and leadership, TOGETHER shows how inclusion can be embedded as a core design feature rather than an add-on. At the same time, our reflections highlight the fragility of these gains within academic and funding systems that often undervalue relational work and rely on short-term staffing and project-based partnerships. If funders, institutions, and commissioners are serious about equity, they must account for the real costs of inclusion and create the structural conditions that allow this work to be sustained. Moving beyond tokenism will require not only better methods, but a shift in how research is funded and valued.

## Supplementary Information

Below is the link to the electronic supplementary material.

Supplementary Material 1

## Data Availability

No datasets were generated or analysed during the current study.
