# Supplementary material for: From tokenism to transformation: lessons from the TOGETHER study for building inclusive and equitable research
Source: Res Involv Engagem. 2026 Mar 30;12:60. doi: 10.1186/s40900-026-00871-y (PMC13162414; doi:10.1186/s40900-026-00871-y)
Supplement: Supplementary file 1 — Supplementary Material 1 [file 40900_2026_871_MOESM1_ESM.docx]

**GRIPP2 Checklist (short form)**

| **GRIPP2-SF Item** | **Addressed in this paper** |
| --- | --- |
| **1. Aim** | This paper aims to provide critical reflections from the TOGETHER study and the key enablers to designing and delivering an inclusive research study in practice. |
| **2. Methods** | The paper idea arose from discussions with the study’s third sector partners and lived experience co-investigator. We used a reflective, retrospective approach drawing on dialogue between researchers (AM, RW, AL, DC, TP), third sector partners (LB, JB), and the co-investigator (KM), with input from Parent Advisory Groups (PAGs) and Community Researchers. AM, LB, and KM co-facilitated two reflective workshops with PAGs, and AM analysed baseline data and five years of meeting minutes. Insights were triangulated and refined collaboratively, ensuring representation from researchers, the third sector, and lived experience in the authorship. The co-authors reflect the research team, co-investigator and third sector partner. |
| **3. Results** | PAGs, the lived experience co-investigator, and the Race Equality Foundation shaped the identification of five key enablers. PAGs and the co-investigator informed findings on lived experience and public involvement, with the co-investigator also helping interpret baseline data. The Race Equality Foundation contributed across all enablers, particularly powering inclusion. Collectively, their contributions underpin the findings and conclusions throughout the paper. |
| **4. Discussion and Conclusions** | Learning and insights from the TOGETHER study were made possible through sustained involvement of PAGs, the lived experience co-investigator, and third sector partners. Our flexible, reflective process—rather than a fixed evaluation—drew on workshops, discussions, baseline data, and meeting minutes. We acknowledge that these reflections are shaped by author positionalities and long-standing relationships, and we offer them as a situated perspective to illuminate practices, tensions, and lessons for inclusive research. |
| **5. Reflections / Critical Perspective** | Although PPI was prioritised within the TOGETHER study, there was no formal plan to evaluate this work so we have offered insights based on reflective and retrospective discussions. We therefore provided a reflective and retrospective account and insights which has involved the key partners and stakeholders including the PAGs, lived experience co-investigator and third sector partner. We embedded PPI as far as possible in the analysis presented in this paper. We are aware that more advanced and prospective planning to evaluate the PPI work may have drawn out additional data and insights and suggest that future research should consider this in future. |
